# Mitigating virus spread through dynamic control of community-based social interactions for infection rate and cost

**DOI:** 10.1007/s13278-022-00953-1

**Published:** 2022-09-09

**Authors:** Ahmad Zareie, Rizos Sakellariou

**Affiliations:** Department of Computer Science, University of Manchester, Manchester, UK

## Abstract

The emergence of a new virus in a community may cause significant overload on health services and may spread out to other communities quickly. Social distancing may help reduce the infection rate within a community and prevent the spread of the virus to other communities. However, social distancing comes at a cost; how to strike a good balance between reduction in infection rate and cost of social distancing may be a challenging problem. In this paper, this problem is formulated as a bi-objective optimization problem. Assuming that in a community-based society interaction links have different capacities, the problem is how to determine link capacity to achieve a good trade-off between infection rate and the costs of social distancing restrictions. A standard epidemic model, Susceptible-Infected-Recovered, is extended to model the spread of a virus in the communities. Two methods are proposed to determine dynamically the extent of contact restriction during a virus outbreak. These methods are evaluated using two synthetic networks; the experimental results demonstrate the effectiveness of the methods in decreasing both infection rate and social distancing cost compared to naive methods.

## Introduction

The emergence of COVID-19 in late 2019 has been accompanied by concerns for unprecedented pressure on public health systems. In order to lessen this pressure and contain the spread of the virus, many countries have adopted strategies, such as social distancing or community interaction restrictions (Flaxman et al. 2020; Chinazzi et al. 2020). It has been argued that social distancing between individuals, by isolation and/or community lockdown, is an effective strategy to decrease infection rate and the number of people affected (Basso et al. 2020; Mayr et al. 2020). Regardless of the effect that such restrictions may have (Bendavid et al. 2021), maintaining social distancing for a long time carries out significant costs in economic and social life, including different aspects such as mental heath, well-being, education, transportation, world trade or manufacturing among others. Thus, imposing social distancing can be a challenging decision for the authorities as they have to assess the trade-off between the benefits of social distancing and its costs (Allen 2022). When making a decision about social distancing some key questions to answer are:

- Which social interactions should be more tightly restricted? When? How tightly?
- Which social interactions could be less tightly restricted? When? How relaxed can they be?

In this paper, a society is modelled as a community-based graph, where a community indicates a group of people, a district, a city or a country; a link between a pair of communities, an inter-community link, denotes the relationship between the individuals of the communities. The nodes inside each community denote the individuals in the community and a link between a pair of nodes, an intra-community link, indicates a relationship between two individuals. The weight of the links indicates the extent of the relationships. The question is how to adjust the capacity of each intra-community and inter-community link during the spreading period of a virus to formulate a social distancing strategy that achieves two goals: decrease infection rate and decrease the cost incurred from social distancing. To address this question, the problem of identifying an optimal limitation factor for each link is formulated as an optimization problem. A Multi-Objective Particle Swarm Optimization (MOPSO) algorithm is used to propose a method that finds an optimal solution.

The contributions of the paper are summarized as follows.

- We model the interactions between individuals in two levels (inter- and intra-community).
- We model the problem as a bi-objective problem where the aim is to consider a trade-off between the benefits and costs of social distancing.
- We apply the MOPSO algorithm to propose a method to solve the problem.

The rest of the paper is organized as follows: Sect. 2 reviews the relevant literature. Interaction and epidemic models are described in Sect. 3; some details of MOPSO are also discussed in this section. The problem is formally defined in Sect. 4. Section 5 describes the details of the proposed method. The results of the experimental evaluation are reported in Sect. 6. Finally, Sect. 7 concludes the paper and suggests some future directions.

## Related work

A number of well-used models exist to understand the diffusion of a virus in a population and predict the number of future infections and/or deaths. Most of these models rely on the popular epidemic model, Susceptible-Infected-Recovered (SIR), initially proposed in Kermack and McKendrick (1927). Describing a Bayesian heterogeneity learning approach, the SIR model is formulated into a hierarchical structure in Hu and Geng (2020). In Goel et al. (2021), a mobility-based variant of the SIR model is introduced to take population distribution and the relationship between different geographical locations into account. The authors in Giordano et al. (2020) consider various stages of infections; they take into account both diagnosed and non-diagnosed infected individuals to account for the role of asymptomatic infection in epidemic spreading. The SIR model is expanded in Calafiore et al. (2020) to propose a time-varying spreading model; this model is applied to understand the changes of rate of infection, death and recovery over time. The interactions between heterogeneous groups of individuals are modelled in Contreras et al. (2020) to propose a multi-group variant of SIR; this model is applied to assess the effectiveness of different public health strategies. An interesting direction to predict infection rate is to analyse the messages exchanged between users in online social networks (Comito 2022; Comito et al. 2018); conversations and social interactions among people about a virus become more frequent when the virus gets more prevalent.

Some research has been conducted to choose good strategies that minimize the spread of a virus. In Bairagi et al. (2020), using location and movement of individuals, a game-theoretic method is proposed to help individuals assess their risk and as a result isolate and/or maintain social distancing. Applying Internet-of-Things technologies, a social distancing detection algorithm is suggested in Ksentini and Brik (2020), which detects and warns people who are not maintaining the minimum recommended social distance. In Hosseini et al. (2020), the epidemic process is modelled as an optimization problem where the goal is to minimize the number of infected countries and slow down worldwide epidemic spread. Other papers, such as Wang et al. (2020), Salathé and Jones (2010), adopt a node immunization strategy to prevent the spread of an epidemic.

Due to the economic costs of social distancing restrictions, there are several studies where cost is considered. The effect of different levels of lockdown (or degree of social distancing restrictions) during an epidemic is analysed in Alvarez et al. (2020), Gonzalez-Eiras and Niepelt (2020); according to their results an optimal level for different time periods during an epidemic may be determined. In Olivier et al. (2020), a predictive model is used to evaluate the impact of varying the level of lockdown and determine an optimal level. The authors in Bosi et al. (2021) focus on an optimal lockdown policy by taking the role of households altruism into account. Acemoglu et al. (2020) determine an optimal level of lockdown which differs for different age-groups (young, middle-aged and old). In Caulkins et al. (2021), the public healthcare system capacity is considered to determine multiple lockdown strategies with different levels and lengths. These papers have a rather static view in their models where the impact of individuals’ travel is not taken into account, something that we consider in our paper.

Individuals travelling (or commuting) between cities or regions may have a significant impact on the spread of the epidemic (Glaeser et al. 2020; Jia et al. 2020). The proliferation of GPS-enabled technologies and location-based social networks can easily provide useful geographic information to model location and mobility patterns of people in a society (Zhao et al. 2016; Stock 2018). In Oum and Wang (2020), a model is presented to analyse the effect of urban traffic congestion on the infection risk and economic costs. The problem of identifying a set of key links between communities to minimize the spread of virus is tackled in Chen et al. (2021). To assess the impact that individuals’ travel may have on spreading a virus, in some studies the relationship between regions is modelled as a graph and the goal is to determine the optimal commuting flow between the regions.

In Birge et al. (2020), a city is modelled as a set of neighbourhoods where each neighbourhood has a number of individuals. Individuals spend a fraction of their time in other neighbourhoods; as they come to contact with individuals from other neighbourhoods, a virus is spread between the neighbourhoods. The goal is to determine the optimal level of lockdown to limit the interaction between each pair of neighbourhoods with minimum costs. Applying linear programming methods, the problem is solved under large and small infection regime scenarios. A similar problem is defined in Fajgelbaum et al. (2020), where commuting locations (such as train stations, workplaces and public places) and the number of commuters between these locations are modelled as a graph. By defining a cost model for the lockdown, the goal is to determine the optimal number of commuters between the locations to curb the spread of a virus with minimum costs. By considering the role of both asymptomatic and symptomatic infected individuals in the spreading process, the problem of identifying an optimal link restriction between communities in order to maintain a trade-off between infection rate and economic costs is discussed in Ma et al. (2020). The research in Birge et al. (2020), Fajgelbaum et al. (2020), Ma et al. (2020) is the closest to our work. The problem formulated in the present paper differs from those defined in Birge et al. (2020), Fajgelbaum et al. (2020), Ma et al. (2020) as, in these papers, only travel of individuals between regions is considered.

In our paper, we model the spread of a virus in two levels (inter- and intra-community) to capture virus propagation between individuals from different regions and also between individuals within the same region. This can lead to an appropriate limitation factor for the links between regions as well as an appropriate level of social distancing restriction within each region; these two may be different. For this propose, the society is modelled as a community-based graph to capture the intra- and inter-community interactions between individuals. The optimal lockdown problem is then defined as a bi-objective optimization problem which considers both the probability of infection and the costs of lockdown. Thanks to the successful Particle Swarm Optimization (PSO) algorithm (Kennedy and Eberhart 1995) that can be used to solve optimization and multi-objective problems (Rahimi et al. 2018; Wang et al. 2019; Cui et al. 2020), PSO is then applied to solve our bi-objective optimization problem under two scenarios: internal-relationship-aware communities where information about the interactions within each community (intra-community interactions) is available, and internal-relationship-agnostic communities where such information is not available. In both scenarios, we assume that information about interactions across communities (inter-community interactions) is available.

## Preliminaries

### Graph modelling

**Fig. 1:**
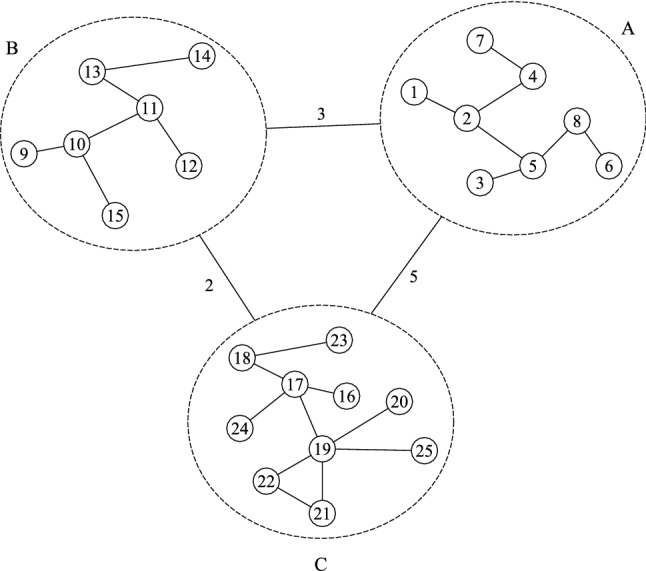
An example graph with inter- and intra-community interactions

The interaction between individuals in a society can be modelled as a graph with communities. Each community (could be a city, a region or a country) indicates a group of individuals. Nodes represent individuals and edges represent the interactions between them. For example, Fig. 1 shows the interaction between individuals in three communities *A*, *B* and *C*. People in a community interact with other people within the same community. In addition, some people from one community travelling to another community interact with some people in the destination community. Accordingly, there are two different types of edges in this model: (i) intra-community edges, which connect two individuals in the same community, like the edge between nodes 4 and 7 in the figure; (ii) inter-community edges, which connect two communities, like the edge between communities *A* and *C*. In practice, inter-community edges may be associated with some sort of transportation, which we assume it is available. It has been suggested that intra-community edges may be determined using tracing apps or smartphones (Zastrow 2020), however, it is not usually trivial to capture all the edges between individuals. Therefore, we consider two situations in our graph: (i) internal-relationship-aware, in which we are aware of the intra-community edges in a community (at least up to two hops from nodes interacted with infected nodes); (ii) internal-relationship-agnostic, in which we are not aware of the intra-community edges in a community.

In essence, a virus may spread from an individual to close contacts (via an intra-community edge) and also from a community to another community (via an inter-community edge). In the proposed model, every edge has a weight to express the capacity of the edge. For an intra-community edge, the weight represents the average percentage of time that two people spend with each other over a period of time. For simplicity, in this paper, we assume that this percentage of time corresponds to the spreading probability between two nodes within the same community. For an inter-community edge, the weight represents the average number of individuals travelling between the communities during a period of time. For instance, in Fig. 1, nodes 2 and 5 interact within community *A* as there is an intra-community edge between them; the inter-community edge between communities *A* and *C* indicates that, on average, 5 individuals travel from community *C* to *A* and 5 individuals from *A* to *C* during a period of time. A higher weight value in an inter-community edge indicates a larger number of passengers travelling between the communities and, accordingly, a greater spreading probability between the communities. Given the weight of the inter-community edges the spreading probability between communities *A* and *C* is greater than the spreading probability between *B* and *C*.

A society is formally modelled as a graph $G(V,E^{V},C,E^{C})$, where the set of nodes $V=\{v_1,v_2,\ldots , v_{|V|}\}$ and the set of communities $C=\{c_1, c_2, \ldots , c_{|C|}\}$ denote the individuals and the communities in the society, respectively. The set of intra-community edges $E^{V}\subset V \times V$ denotes the interactions between the individuals. If there is an interaction between two nodes $v_i$ and $v_j$, they are called neighbours or friends; this is shown by the edge $e^{V}_{ij}$. A weight $w^{V}_{ij}$ is assigned to this intra-community edge, which, as already mentioned, indicates the spreading probability between nodes $v_i$ and $v_j$ when one of the nodes is infected. The set of inter-community edges $E^{C}\subset C \times C$ indicates interactions between communities. If there are individuals travelling between two communities $c_i$ and $c_j$ then there is an inter-community edge $e^{C}_{ij}\in E^{C}$; a weight $w^{C}_{ij}$ is assigned to this inter-community edge to indicate the number of individuals travelling between the two communities during a period of time. To keep things simple, in the rest of paper, we use $e_{ij}$ without superscript to refer to an edge in $E^{V}$ or $E^{C}$. We use $v^{C}_i$ to indicate the community, which $v_i$ belongs to. In the interaction-agnostic scenario, we have no information about the set of intra-community edges, $E^{V}$, and the only available information about this set is the average weight of the edges (essentially, the average time that people spend together in each community).

### Spreading model

The Susceptible-Infected-Recovered (SIR) model (Kermack and McKendrick 1927) is a widely used epidemic model that emulates the spread of a virus. In this model, each node can be in one of susceptible (*SU*), infected (*IN*), or recovered (*RE*) states. At timestamp $t=0$, initially infected nodes are set to *IN* and all other nodes are set to *SU*. In each timestamp $t>0$, each infected node $v_i$ goes to recovered state with probability $\gamma$ after its attempt to infect each of its susceptible neighbours. The spreading process continues until no infected nodes exist.

In this paper, in order to take into account the role of communities and individuals travelling in the spreading process, the SIR model is extended as follows. It is supposed that a set of infected nodes is detected in a society at the beginning of the process. Some individuals, or exposed nodes, have been in contact with these infected ones. It is also supposed that all exposed nodes cannot be isolated or tested (this could be because of the cost of isolation or the shortage of tests or both). Exposed nodes are infected based on the probability related to the weight of the edge between them and initially infected neighbours. The exposed nodes infected by their neighbours are set to *IN* at timestamp $t=0$; all other nodes are set to susceptible state *SU*. At each timestamp $t>0$, the virus can spread from an infected node to a number of neighbours. Here, we suppose that at each timestamp, the virus can spread up to two hops from infected nodes; this means that at each timestamp just first and second order neighbours of an infected node can be affected. Two types of spreading may occur: (i) inter-community spreading, in which the virus may spread between a pair of connected communities $c_i$ and $c_j$ on the basis of the weight $w^{C}_{ij}$; (ii) intra-community spreading, in which each node $v_i\in IN$ infects each of its susceptible intra-community neighbours $v_j$ with probability $w^{V}_{ij}$. The infection process in each timestamp is described in detail next.

At each timestamp $t>0$, in order to simulate inter-community spreading from each community $c_i$ to $c_j$, $i \ne j$, first, a set $Psg_{ij}$ containing $w^{C}_{ij}$ nodes is randomly selected from community $c_i$ to travel to community $c_j$. For each node $v_r \in Psg_{ij}$, a number $\alpha$ of the nodes in community $c_j$ are randomly selected as $v_r$’s inter-community neighbours; these neighbours are temporary neighbours just for the current timestamp. Due to $v_r$’s commuting, the edges between this node and its intra-community neighbours are temporarily removed in the first iteration. In the first iteration of the timestamp, each infected node may infect each of its susceptible intra-community or inter-community neighbours; then it goes to recovered status based on a recovering probability $\gamma$. The infection probability for each inter-community neighbour in community $c_j$ is the average of the weights of the edges in the community. It is supposed that all commuters return to their communities in the second iteration of the timestamp; so inter-community neighbours are removed and intra-community neighbours are restored for each commuter node $v_r$. Each node infected in the first iteration may infect each of its intra-community neighbours likewise in the first iteration. At the end of each timestamp, each infected node is detected (recovered or quarantined) based on the recovering probability $\gamma$. All nodes in contact with detected nodes are considered as potentially infected nodes in the next timestamp. The spreading process continues in successive timestamps until no infected nodes remain. For simplicity, we consider $\gamma =1$, but the proposed equations could be extended to consider any value of $\gamma$.

### Multi-objective optimization problems

A multi-objective optimization problem (MOP) is the problem of finding a set of decision variables to optimize a vector of objective functions which are usually in conflict with each other. A minimization MOP is modelled as a set of objective functions $\hbox {min}_{a\in A} F(a)=\hbox {min}_{a\in A}\{f_1(a),f_2(a),\ldots ,f_z(a)\}$ which must be simultaneously minimized; *A* is a set of feasible solutions and $f_i(a)$ is the value of $i^{ th}$ objective function of solution *a*. A solution $a^{(1)} \in A$ dominates another solution $a^{(2)}\in A$, denoted by $a^{(1)}\succ a^{(2)}$, if

$$\begin{aligned}&\forall i \in (1,\ldots ,z) \quad f_i\left(a^{(1)}\right)\le f_i\left(a^{(2)}\right)\; \wedge \; \exists i \in (1,\ldots ,z) \\&\quad f_i\left(a^{(1)}\right)<f_i\left(a^{(2)}\right) \end{aligned}$$

If neither of these solutions dominates another one, it is denoted by $a^{(1)}\sim a^{(2)}$. MOP typically aims to determine a set of Pareto solutions; $a^{(*)} \in A$ is a Pareto solution if there is no solution $a\in A$ that $a\succ a^{(*)}$. A set of Pareto solutions, called Pareto set or non-dominated solution set, is defined as

$$\begin{aligned} PS=\left\{a^{(*)}\in A \; | \; \not \exists a\in A \wedge a\succ a^{(*)}\right\}. \end{aligned}$$

In order to determine the optimal solution among the solutions in *PS*, a multi-criteria decision-making (MCDM) approach can be applied. Vlsekriterijumska Optimizacija I Kompromisno Resenje (VIKOR) (Opricovic and Tzeng 2004) is one of the MCDM approaches which has been used in different problems (Gul et al. 2016). In this paper, we apply the VIKOR approach to find the personally best and globally best solutions in each iteration. For this purpose, VIKOR determines the ideal solution which gives the best value for each objective among all Pareto solutions, it assigns a weight to the objectives and selects the Pareto solution with the closest distance to the ideal solution as the optimal solution; this is considered as the best trade-off between the objectives. From the different strategies used by VIKOR to determine the weight of objectives, we choose entropy (Gul et al. 2016). In addition to the weight given to the objectives by VIKOR, we can manually indicate the relative importance of the objectives if needed. In this case, the weight of objective function $f_i$ ($i=1,2$) can be calculated as $w_i={(h_i\cdot q_i)}/{\sum _{j=1}^2 (h_j\cdot q_j)}$, where $h_i$ denotes the weights calculated by VIKOR for objective $f_i$ and $q_i$ denotes any manually chosen weights for objective $f_i$. Clearly when for all objectives $q_i$ is the same, the weights are totally determined by VIKOR.

In this paper, the Particle Swarm Optimization (PSO) algorithm (Kennedy and Eberhart 1995) is applied to search the problem space and determine a set of feasible solutions and, subsequently, a set of Pareto solutions. PSO is a metaheuristic algorithm inspired by the behaviour of a bird flock searching for corns. In PSO, birds in a flock with population size *n* are defined as a swarm of *n* particles; each bird is modelled as a particle $P_i(X_i,V_i)$ that corresponds to a solution of the problem. $X_i=\{x_{i1},x_{i2},\ldots x_{ik}\}$ is a position vector denoting the current position of the particle; $V_i=\{v_{i1},v_{i2},\ldots v_{ik}\}$ is a velocity vector providing the direction of the particle to adjust particle movement towards the optimal solution. The number of variables of the problem is denoted by *k*. Particles update their position and velocity vectors to search the problem space iteratively. The following rules are applied in each iteration to update the vectors of particle *i*:

1 $$\begin{aligned} \begin{aligned} V_i&=\omega V_i+c_1r_1(PB_i-X_i)+c_2r_2(GB-X_i) \\ X_i&=X_i+V_i, \end{aligned} \end{aligned}$$

where $\omega$ is inertia weight; $PB_i=\{PB_{i1},PB_{i2},\ldots PB_{ik}\}$ is the personally best position of particle *i* in previous iterations, and $GB_i=\{GB_{i1},GB_{i2},\ldots GB_{ik}\}$ represents the globally best position in the swarm in previous iterations. The parameters $c_1$ and $c_2$ denote learning factors, and $r_1$ and $r_2$ denote random values $\in [0,1]$. The particles search the problem space in *gmax* iterations and the globally best position found during the iterations is returned as the optimal solution.

## Problem definition

Minimizing the spread of a disease is an important issue to mitigate its consequences and impact to a society. Minimizing interaction between people can be an effective way for this goal. Thus, in this paper, we aim to identify a set of critical links to contain the spread of disease over time. Two different containment strategies are adopted in this paper: (i) limit the interaction between a pair of neighbours by imposing limitations to the edge between the pairs; (ii) limit the interaction between a pair of communities by imposing limitations to the inter-community edge between the pairs. In fact, we aim to limit the critical intra-community edges by limiting the time that two neighbours spend together and the critical inter-community edges by limiting the number of individuals travelling between the communities. It is noted that imposing limitations on the edges has some cost. In addition, the edges cannot be limited for a long time, so we dynamically limit and unlimit the edges (i.e. tighten and relax the restrictions) over the spreading period. That is to say, at each timestamp we decide to limit or unlimit a set of edges (intra- and inter-community).

The cost of limitation for each edge depends on its weight. For example, if the time that two friends spend together is long they are more reluctant to be banned from meeting each other; a blanket ban may have a high cost. Thus, suppose that limiting $\eta _{ij} \%$ of the interaction between two neighbours $v_i$ and $v_j$ (connected by the edge $e^{V}_{ij}$) for one timestamp costs $LC^{V}_{ij}= w^{V}_{ij}\cdot \eta _{ij}$. Again, suppose that limiting $\eta _{ij} \%$ of the interaction between two communities $c_i$ and $c_j$ (connected by the edge $e^{C}_{ij}$) for one timestamp costs $LC^{C}_{ij}=w^{C}_{ij}\cdot \eta _{ij}\% \cdot 2 \cdot \alpha$. The weight of each inter-community edge represents the number of commuters in both directions. As each commuter may have $\alpha$ inter-community neighbours, we consider $2\cdot \alpha$ to determine the cost of limiting inter-community edges. According to these definitions, every edge can be stated as $\eta \%$-limited at each timestamp during the spreading process, where $\eta$ is the limitation factor of the edge, a value in [0, 1]; if $\eta =0$ the edge is unlimited, $0<\eta <1$ the edge is limited and if $\eta =1$ the edge is completely blocked.

We call this problem *Progressive Limitation of Critical Interactions* (PLCI). The goal is to choose an optimal $\eta _{ij}$ for every edge $e_{ij}$ (intra- and inter-communities) at each timestamp *t* during the spreading process. Thus, the PLCI problem is defined as: given a society modelled by a graph $G(V,E^{V},C,E^{C})$ and a set of nodes in contact with infected nodes, the goal is to determine a limitation factor $\eta _{ij}$ for every edge $e_{ij}$ at each timestamp *t* so that the number of infected nodes is minimized with a minimum cost. That is to say, the goal is to find the trade-off between the number of infected individuals and the cost of limitations on the edges. Suppose that the spreading process continues for *T* timestamps. Then, as a formal definition, the problem aims to determine the limitation factor for every edge at each timestamp $t=1\ldots T$ to set $E^{V}(t)$ and $E^{C}(t)$, where $w_{ij}(t)=w_{ij}\cdot \eta _{ij}$. The objective functions of the problem in each timestamp *t* are defined as follows:

- function $f_1(E^{V}(t),E^{C}(t))$ denotes a nodes’ infection probability reduction in timestamp *t*. That is to say, to what extent the infection probability of nodes decreases in the graph with edges $E^{V}(t)$ and $E^{C}(t)$ compared to the graph with edges $E^{V}$ and $E^{C}$. The value of $f_1$ in timestamp *t* is calculated using Eq. (2):
  2 $$\begin{aligned} f_1(E^{V}(t),E^{C}(t))=\underset{v_d \in V}{\sum }\frac{I_d-{I_d(t)}}{I_d}, \end{aligned}$$
  where $I_d$ indicates the infection probability of node $v_d$ in the graph with edge sets $E^{V}$ and $E^{C}$; ${I_d(t)}$ indicates this probability in the graph with edge sets $E^{V}(t)$ and $E^{C}(t)$. Maximizing the function $f_1$ at each timestamp *t* leads to minimizing the number of infected individuals at the end of the spreading process.
- function $f_2(E^{V}(t),E^{C}(t))$ indicates the cost of limiting the edges during timestamp *t*, which is calculated using Eq. (3):
  3 $$\begin{aligned} f_2\left(E^{V}(t),E^{C}(t)\right)=\underset{e^{V}_{ij} \in E^{V}}{\sum }{LC^{V}_{ij}} + \underset{e^C_{ij} \in E^{C}}{\sum }{LC^{C}_{ij}} \end{aligned}$$
  The first part of the equation corresponds the cost of the limitation of intra-community edges and the second part corresponds the cost of the limitation of inter-community edges.

The goal of the PLCI problem at each timestamp *t* is defined as a multi-objective problem in Eq. (4):

4 $$\begin{aligned} \hbox {PLCI}(t)=\underset{E^{V}(t) , E^{C}(t)}{\hbox {min}}\left\{ -f_1(E^{V}(t),E^{C}(t)),\, f_2(E^{V}(t),E^{C}(t))\right\} . \end{aligned}$$

The overall framework to address the problem is shown in Algorithm 1. Due to the detection of infected people, a set of exposed individuals who have been in contact with infected nodes is determined as potentially infected individuals in line 1. Edge limitation continues in sequential timestamps *ts* while there are potentially infected individuals in a society, i.e. $PT\ne \emptyset$. In line 6, the capacity of the edges is determined and some edges are limited or unlimited according to the infection status in the society. In lines 8–14, the individuals who commute between communities and their temporary neighbours (inter-community neighbours) are determined. A set of potentially infected nodes is randomly determined as infected nodes in line 17. The first iteration of the spreading process is simulated in lines 18–22 where the virus can spread through intra- and inter-community edges. In lines 24–25, the commuted individuals return back to their communities. The second iteration of spreading is simulated in lines 27–31. In lines 33–36, the infected nodes are recovered and the set of nodes who were in contact with them are set to be exposed individuals (potentially infected nodes). This process continues until no potentially infected nodes remain. In the next section, a method to determine the limitation factor of the edges in line 6 of the framework is proposed.
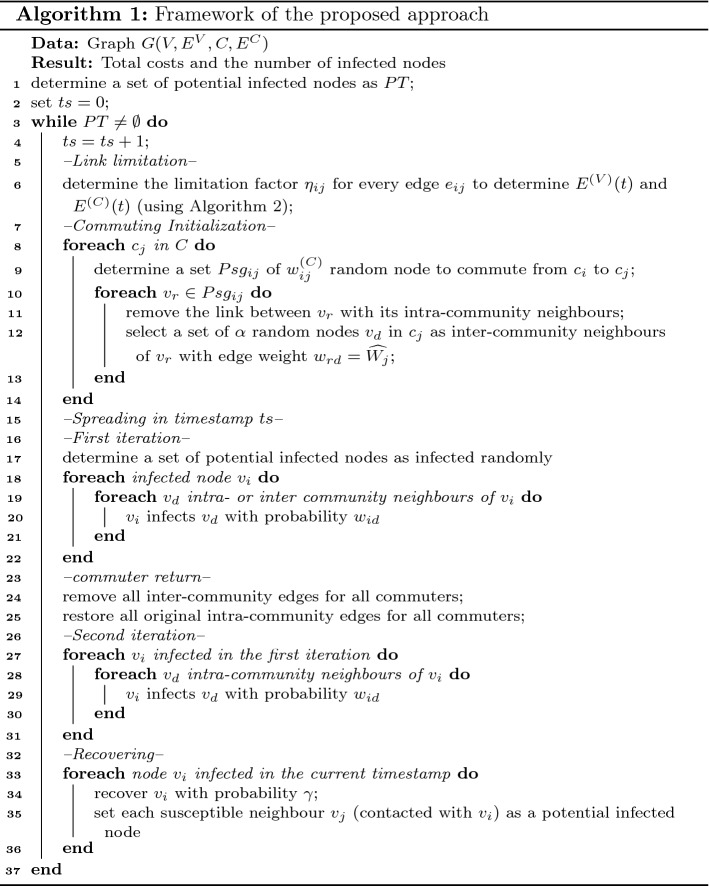


## A proposed solution

In Sect. 5.1, we propose a method to determine an optimal value for the limitation factor of every edge at each timestamp $t>0$. For this purpose, the infection probability of each node in each timestamp needs to be calculated. Section 5.2 describes the details on how to calculate the infection probability of each node using two scenarios: internal-relationship-aware and internal-relationship-agnostic.

### Determining the limitation factor of links

In this section, we propose a multi-objective method, based on PSO, to find an optimal solution of the PLCI problem, defined in Eq. (4). This method has two variants: structure-aware-edge-limitation (SAEL) and structure-unaware-edge-limitation (SUEL), which solve the problem for the internal-relationship-aware and internal-relationship-agnostic scenarios, respectively.

In this method, first a set of critical edges is determined. Then, the limitation factor for each critical edge is determined using PSO; the limitation factor for all other edges is set to 0 (this means that all non-critical edges are unlimited). The number of critical edges is denoted by *k*; also, $k_1$ and $k_2$ express the number of intra- and inter-community critical edges, respectively, hence $k=k_1+k_2$. In our method, each particle *i* contains a position vector $X_i$ and a velocity vector $V_i$. The vector $X_i$ corresponds to a solution of the particle and is modelled as an array of *k* genes, from which $k_1$ genes relate to intra-community critical edges and $k_2$ genes relate to inter-community critical edges. The value in each gene, which is in [0, 1], indicates the limitation factor of a critical edge.
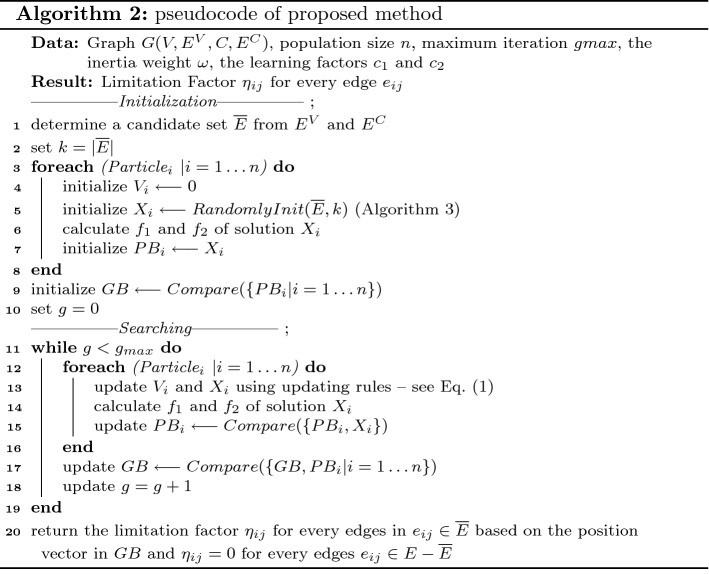


Details of the proposed method to determine the limitation factor of each edge (for both SAEL and SUEL) at each timestamp are given in Algorithm 2. In lines 1–2, a set of intra- and inter-community edges is determined as critical edges for some limitation; the cardinality of this set is *k*. The intra-community edges connected to the exposed individuals and the inter-community edges connected to the communities with at least one exposed individual are considered as critical edges. In lines 3–8, a set of *n* particles is initialized; for each particle, the velocity vector is set to 0 and the position vector is generated randomly using the function RandomlyInit (see Algorithm 3). The value of the objective functions $f_1$ and $f_2$ of the particle is calculated in line 6; the personally best position of the particle is set to its initial position in line 7. In line 9, function Compare(PB) is applied to determine the optimal solution as the globally best position. This function gets a set PB of feasible solutions; it first determines a set of Pareto solutions in the solution set PB. Then, if the Pareto set has just one non-dominated solution, this solution is selected as an optimal solution; otherwise the VIKOR approach is applied to select an optimal solution. In lines 11–18, the algorithm iterates for $g_\mathrm{max}$ iterations to update the position of the particles and search the problem space. The globally best solution GB is returned as the problem solution in line 20. The pseudocode of function RandomlyInit is shown in Algorithm 3.
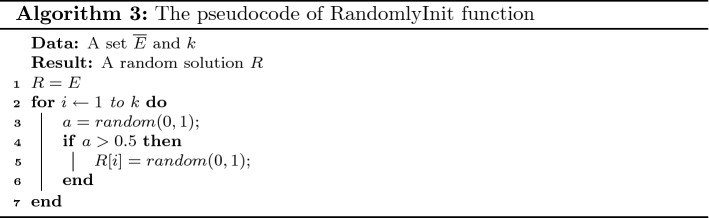


In order to calculate the value of $f_2$ in lines 6 and 14 of Algorithm 2, Eq. (3) is applied. The next section describes how to calculate the value of $f_1$ in lines 6 and 14 of Algorithm 2.

### Infection probability

There are two possible ways for a susceptible node $v_d$ in community $c_j$ to be infected in a timestamp: (i) $v_d$ stays in community $c_j$ and may be infected by an intra-community neighbour or by an inter-community neighbour commuting from another community $c_i$ to $c_j$; (ii) node $v_d$ commutes to another community $c_i$ and may be infected by an inter-community neighbour in $c_i$. As mentioned in Algorithm 1, we consider two iterations in each timestamp so a node may be infected through one or two-hop paths in the timestamp. The infection probability of $v_d$ through one- or two-hop paths is determined as follows.

Recall that $w^{C}_{ij}$ denotes the number of nodes commuting from $c_i$ to $c_j$ and vice versa in a timestamp. Therefore, the probability that each node $v_d$ in $c_j$ commutes to another community is $r_j=\sum _{c_i \in C}\frac{w^{C}_{ij}}{|c_j|}$, where $|c_j|$ indicates the number of nodes in community $c_j$. The infection probability of $v_d$ trough one-hop paths is calculated using Eq. (5):

5 $$\begin{aligned} \rho _{j}^{(1)}(v_d)= P_{j}^{(st)}(v_d)+P_{j}^{(cm)}(v_d), \end{aligned}$$

where $P_{j}^{(st)}$ denotes the probability that $v_d$ is infected when it stays in its community and $P_{j}^{(cm)}$ indicates the probability that $v_d$ is infected when it commutes to another community. Due to the assumption that commuting happens in the first iteration in each timestamp, the infection probability in the second iteration relates only to intra-community neighbours. The infection probability of node $v_d$ through the paths up to two hops is determined as:

6 $$\begin{aligned} \rho _{j}^{(2)}(v_d)&= 1-\left( \left(1-\rho _{j}^{(1)}(v_d)\right)\right. \\ &\quad\left. \cdot \prod _{v_q \in {\mathcal {N}}_d\; \wedge \;v_q \notin PT}\left(1-\rho _{j}^{(1)}(v_q)\cdot w^{V}_{qd}\right)\right) , \end{aligned}$$

where $(1-\rho _{j}^{(1)}(v_d))$ denotes the probability that $v_d$ is not infected by one-hop paths. The notations ${\mathcal {N}}_d$ and *PT* indicate the intra-community neighbours set of $v_d$ and the potentially infected set. The infection probability of each susceptible node $v_d$ during a timestamp is determined by Eq. (6). In what follows, we describe how the values of $P_{j}^{(st)}(v_d)$ and $P_{j}^{(cm)}(v_d)$ in Eq 5 are calculated.

(i) *When a node* $v_d$ *stays in its community*: In this case, the node may be infected by potentially infected intra-community neighbours or by potentially infected inter-community neighbours, commuting from another community to $c_j$. Thus, $P_{j}^{(st)}$ is calculated using Eq. (7):

7 $$\begin{aligned} P_{j}^{(st)}(v_d)&= (1-r_j)\cdot \left( 1- \prod _{v_q \in {\mathcal {N}}_d \wedge v_q \in PT}\left( 1-w^{V}_{qd}\cdot (1-r_{j})\right) \right. \\ &\quad\left. \cdot \prod _{c_i \in C}\left(1-\rho _{ij}^{(c)}\right) \right) , \end{aligned}$$

where $(1-r_{j})$ is the probability that $v_d$ stays in its community $c_j$ in the timestamp and $\prod _{v_q \in {\mathcal {N}}_d \wedge v_q \in PT}(1-w^{V}_{qd}\cdot (1-r_{j}))$ calculates the probability that $v_d$ is not infected by intra-community neighbours. It is noted, that a potentially infected intra-community neighbour of $v_d$ may commute to another community in the timestamp, so the infection of $v_d$ by this neighbour depends on the staying of the neighbour in community $c_j$, which is $(1-r_{j})$. The notation $(1-\rho _{ij}^{(c)})$ is used for the probability that $v_d$ is not infected by inter-community neighbours; whereas $\rho _{ij}^{(c)}$ is the infection probability of each node in $c_j$ by inter-community neighbours commuting from community $c_i$ to $c_j$. How to calculate $\rho _{ij}^{(c)}$ is described next.

Let denote the total number of nodes and the expected number of infected nodes in community $c_i$ by $N_i$ and $n_i$, respectively (the expected number of infected nodes in a community is determined as the sum of the infection probability of all potentially infected nodes in the community). The probability that a number of nodes *l* commuting from $c_i$ to $c_j$ are infected is calculated using Eq. (8). In this equation, $$\left( {\begin{array}{c}Y\\ X\end{array}}\right)$$ is the number of combinations of *X* elements out of *Y* elements, which is calculated as $\frac{Y!}{X!\cdot (Y-X)!}$.

8 $$\begin{aligned} A_{ij}^{(l)}=\frac{\left( \begin{array}{c}{n_i}\\ {l}\end{array}\right) \cdot \left( \begin{array}{c}{N_i-n_i}\\ {w^{(C)}_{ij}-l}\end{array}\right) }{\left( \begin{array}{c}{N_i}\\ {w^{(C)}_{ij}}\end{array}\right) } \end{aligned}$$

The probability that a number *f* of these *l* infected commuters visit node $v_d$ (i.e. the number of potentially infected inter-community neighbours of $v_d$ who commute from $c_i$ to $c_j$) is calculated using Eq. (9):

9 $$\begin{aligned} B_{ij}^{(l,f)}=\left( {\begin{array}{c}l\\ f\end{array}}\right) \cdot \delta ^f\cdot (1-\delta )^{l-f}, \end{aligned}$$

where $\delta =\frac{\alpha }{|c_j|}$ is the probability that a node commuting from $c_i$ to $c_j$ visits node $v_d$. Given Eqs. (8) and (9), the probability that node $v_d$ has *f* potentially infected inter-community neighbours with different values of *l* is calculated using Eq. (10):

10 $$\begin{aligned} P_{ij}^{(f)}=\sum _{l=f}^{w^{(C)}_{ij}} A_{ij}^{(l)} \cdot B_{ij}^{(l,f)} \end{aligned}$$

Finally, the infection probability of each node $v_d$ in community $c_j$ by inter-community neighbours commuting from $c_i$ to $c_j$ is calculated as:

11 $$\begin{aligned} \rho _{ij}^{(c)}=\sum _{f=1}^{w^{(C)}_{ij}} \left(1-\left(1-\widehat{W_j}\right)^f\right)\cdot P_{ij}^{(f)}, \end{aligned}$$

where $\widehat{W_j}$ is the average of the weight of the edges in community $c_j$.

(ii) *When a node* $v_d$ *commutes from community* $c_j$ *to another community* $c_i$: In this case, the node may be infected by potentially infected inter-community neighbours in $c_i$. This probability, denoted by $P_{j}^{(cm)}$, is determined using Eq. (12), where $\widehat{W_j}$ is the average weight of the edges in community $c_j$ and $\frac{w^{(C)}_{ij}}{N_j}$ is the probability of a node $v_d$ commuting from $c_j$ to $c_i$.

12 $$\begin{aligned} P_{j}^{(cm)}=1-\prod _{c_i\in C}\sum _{f=1}^{w^{(C)}_{ij}} \left( \left(1-\left(1-\widehat{W_i}\right)^f\right)\cdot A_{ij}^{(f)}\cdot \frac{w^{(C)}_{ij}}{N_j}\right) \end{aligned}$$

As mentioned in Sect. 2, in the internal-relationship-agnostic scenario the information about the structure of the edges inside the communities is not always available. Thus, Eqs. (6) and (7) cannot be calculated for the structure-unaware-edge limitation (SUEL) method. So, we propose an alternative solution for SUEL, where Eq. (6) is replaced as follows. Given the equation discussed in Sect. 3.2, in the first iteration of each timestamp, the expected number of infected nodes in $c_j$ that are infected by other nodes in $c_j$ is calculated according to Eq. (13), where $\widehat{D_j}$ and $s_j$ are the average degree of the nodes and the number of susceptible nodes in community $c_j$, respectively. The notation $\widehat{W_j}\cdot \widehat{D_j}$ indicates the number of neighbours that can be infected by an infected node.

13 $$\begin{aligned} \hbox {IE}_j=\frac{\widehat{W_j}\cdot \widehat{D_j}\cdot S_j \cdot n_j }{N_j} \end{aligned}$$

Therefore, Eq. (7) in SUEL is replaced by Eq. (14):

14 $$\begin{aligned} \hbox {Ag}P_{j}^{(st)}(v_d)=\left( 1- \frac{\hbox {IE}_j}{S_j} \cdot \prod _{c_i \in C}\left( 1-\rho _{ij}^{(c)}\right) \right) \cdot (1-r_j) \end{aligned}$$

As a result of the recovery of infected nodes in the first iteration of each timestamp, the number of nodes remaining in community $c_j$ is $N^{(2)}_j=N_j-n_j$. The number of nodes infected in the first iteration (i.e. $\hbox {IE}_j$) determines the expected number of the infected nodes in community $c_j$ in the second iteration. Also, the expected number of susceptible nodes is $s^{(2)}_j=S_j-\hbox {IE}_j$. Therefore, the expected number of nodes in $c_j$, which may be infected by other nodes of the community in the second iteration, is calculated by Eq. (15):

15 $$\begin{aligned} \hbox {IE}^{(2)}_j=\frac{\widehat{W_j}\cdot \widehat{D_j}\cdot s^{(2)}_j \cdot IE_j }{N^{(2)}_j} \end{aligned}$$

In order to calculate the infection probability of node $v_d$ through the paths up to two hops in SUEL, Eq. (6) is replaced by Eq. (16):

16 $$\begin{aligned} \hbox {Ag}\rho _{j}^{(2)}(v_d)=1-\left( \left( 1-\rho _{j}^{(1)}(v_d)\right) \cdot \left( 1-\frac{\hbox {IE}^{(2)}_j}{s^{(2)}_j}\right) \right) \end{aligned}$$

Algorithm 4 shows pseudocode for the calculation of the infection probability of every node in the network. It returns an array *I* with |*V*| entries, where $I_d$ indicates the infection probability of node $v_d$.
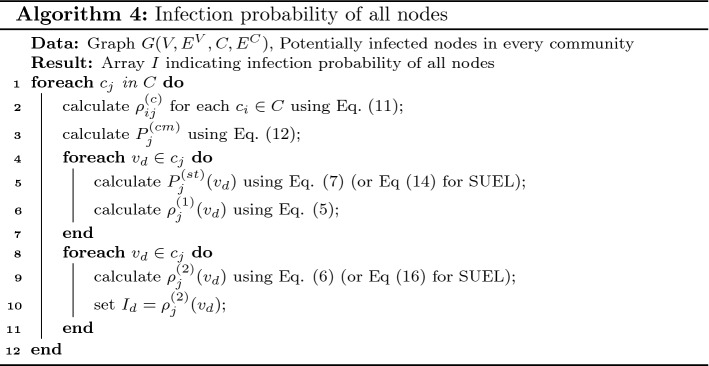


## Experimental evaluation

In this section, the performance of the proposed methods is evaluated. To do so, a set of experiments is carried out and the results obtained by the proposed methods SAEL and SUEL are compared to the simple heuristics described below:

- The baseline approach is where none of the edges is limited; this approach is called No-Intervention (NINR) in the rest of the paper.
- The edges with greatest weights are blocked to minimize the spread of virus; this approach is called Max-Weight (MXW) in the rest of the paper.
- To take into account the cost of edge limitation, the edges with smallest weights are blocked to minimize the spread of virus (this allows blocking of a relatively large number of edges); this approach is called Min-Weight (MNW) in the rest of the paper.

**Fig. 2:**
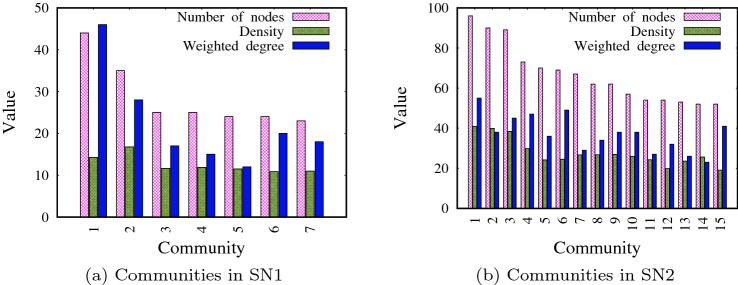
The characteristics of communities in SN1 and SN2

In order to generate a set of networks for evaluation, we apply the Lancichinetti–Fortunato–Radicchi (LFR) benchmark (Lancichinetti et al. 2008), which is used to generate synthetic networks based on: number of nodes |*V*|, average degree of nodes $\langle d \rangle$, minimum community size *m*(*C*), maximum community size *M*(*C*) and mixing parameter $\mu$. The mixing parameter $\mu$ defines the expected proportion of edges which connect two nodes in different communities. Two synthetic networks with varying features, SN1 and SN2, are generated. We set $|V|=200$, $\langle d \rangle =5$, $m(C)=20$, $M(C)=40$ and $\mu =0.10$ to generate SN1. We set $|V|=1000$, $\langle d \rangle =8$, $m(C)=50$, $M(C)=100$ and $\mu =0.12$ to generate SN2. To determine the inter-community edges in SN1 and SN2, we first check for edges connecting any node in community $C_i$ to any node in community $C_j$. If there are *x* such edges, we remove these edges between nodes and we create an inter-community edge between communities $C_i$ and $C_j$ with weight *x*. The characteristics of SN1 and SN2 are shown in Table 1. The table shows the number of nodes (|*V*|), the number of intra-community edges ($|E^V|$), the average weight of the intra-community edges ($\langle W^V \rangle$), the number of communities (|*C*|), the number of inter-community edges ($|E^C|$), the average weight of the inter-community edges ($\langle W^C\rangle$), the number of nodes in the biggest community (*M*(*C*)) and the number of nodes in the smallest community (*m*(*C*)). Furthermore, the characteristics of each community, for both SN1 and SN2, are shown in Fig. 2; these characteristics include the number of nodes in the community, density, which is the sum of the weight of edges between the nodes (intra-community edges) within the community, and the weighted degree, which is the sum of the weight of edges between the community and other communities (inter-community edges).

**Table 1:** Characteristics of the two synthetic networks used in the experiments

Network	|*V*|	$|E^V|$	$\langle W^V\rangle$	|*C*|	$|E^C|$	$\langle W^C\rangle$	*M*(*C*)	*m*(*C*)
SN1	200	613	0.1434	7	19	4.1053	44	23
SN2	1000	2208	0.1886	15	98	2.8469	96	52

Four different experiments are conducted to assess the performance of the proposed methods.

- In the first experiment, the effect of varying the size of population (*n*) and the number of iterations (*gmax*) in Algorithm 2 on the final value of the objective functions (*f*1 and *f*2) is considered to select an appropriate value for *n* and *gmax*.
- In the second experiment, the Pareto solution sets provided by the proposed methods are examined to assess the capability of the methods in searching the problem space.
- The third experiment considers the number of infections as well as the cost of limitations in different timestamps as the virus spreads.
- Finally, the impact of the proposed methods in minimizing spread of an infection is assessed for different values of the number of inter-community neighbours ($\alpha$) in the fourth experiment.

Each experiment is repeated 50 times with a different initial set of infected nodes selected randomly. For each set, the extended SIR model is independently repeated 100 times to increase confidence in the obtained results. In the first three experiments, we set $\alpha =3$, which means that each individual commuting from one community to another community has three inter-community neighbours in the destination community. For the parameters $\omega$ (inertia weight), $c_1$ and $c_2$ (learning factors) in the proposed methods (see Algorithm 2), we follow the suggestions in Eberhart and Shi (2000), Shi and Eberhart (1999), Ratnaweera et al. (2004). Thus, the value of $\omega$ varies from 0.9 to 0.4, the value of $c_1$ varies from 2.5 to 0.5 and the value of $c_2$ varies from 0.5 to 2.5. Finally, for the second and third experiments, different values of $q_1$ and $q_2$ are used to evaluate the performance of the proposed methods when the objectives may not have the same importance; in experiments one and four, $q_1$ and $q_2$ are both set 1.

### Experiment 1

**Fig. 3:**
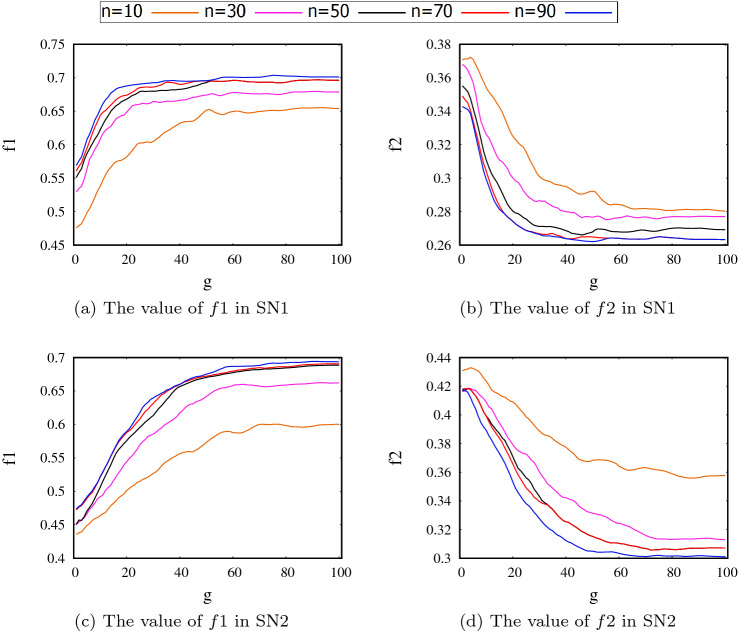
The value of objective functions in different iteration *g* for different population size *n*

To select an appropriate value for the number of iterations *gmax* and population size *n*, we consider the solution provided by the proposed method SAEL for a range of different values of *n* and iterations *g*. In each case, we report the value for each objective, $f_1$ and $f_2$. The results for both networks SN1 and SN2 are shown in Fig. 3. It can be seen that as the number of iterations *g* exceeds 50, the value of the objective functions do not change significantly. Thus, in order to achieve a trade-off between effectiveness and efficiency, the value of *gmax* is set to 50 in the next experiments. Furthermore, the figure shows that the value of the objective functions improves as the population size increases. However, no significant improvement is obtained when the population size takes values higher than 50. Thus, in order to achieve a trade-off between effectiveness and efficiency, the size of the population is fixed at 50 in both SAEL and SUEL methods for the following experiments.

### Experiment 2

**Fig. 4:**
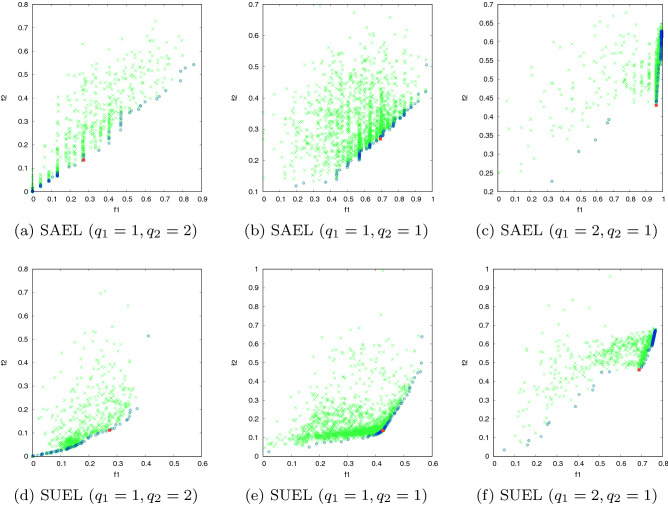
The search space of the proposed methods in SN1: feasible solutions (green) Pareto solutions (blue) and optimal solution (red)

**Fig. 5:**
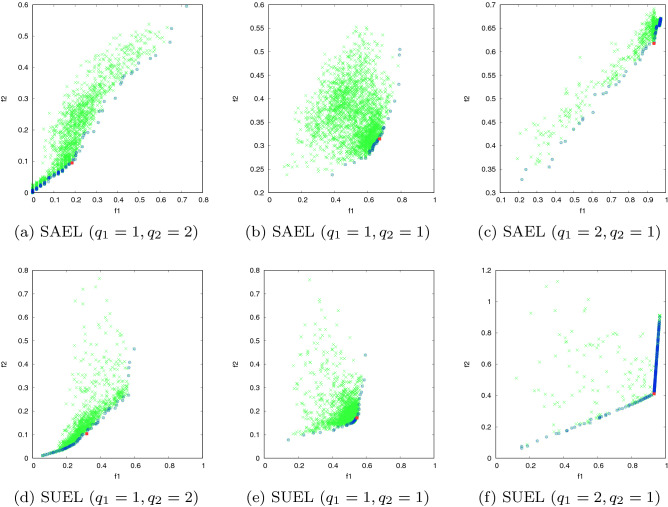
The search space of the proposed methods in SN2: feasible solutions (green) Pareto solutions (blue) and optimal solution (red)

In this experiment the solutions provided by the proposed methods in a timestamp are evaluated to assess the diversity of the solutions and the suitability of the methods to search the problem space. For this purpose, we consider different optional relative importance ($q_1$ and $q_2$) of objective functions to determine weights $w_1$ and $w_2$ for the importance of the objective functions $f_1$ and $f_2$ (defined in Sect. 4), respectively. The solution for both SAEL and SUEL methods with different values of $q_1$ and $q_2$ on network SN1 and SN2 is shown in Figs. 4 and 5, respectively. The feasible solutions, Pareto solutions and optimal solutions provided by proposed methods are represented by the green, blue and red points, respectively. Recall that the goal is maximizing $f_1$ and minimizing $f_2$, so the closer a solution is to the bottom right of each graph, the closer to the optimal solution is. The results show diversity of the feasible solutions and overall good suitability of the proposed methods to examine the search space of the problem. It can be seen that for different importance of the objective functions, the proposed methods try to search different spaces of the problem. A comparison of the solutions provided by SAEL and SUEL reveals that, due to the availability of the structural relationships inside the communities, SAEL provides solutions with a better trade-off between the objectives than the solutions provided by SUEL.

### Experiment 3

**Fig. 6:**
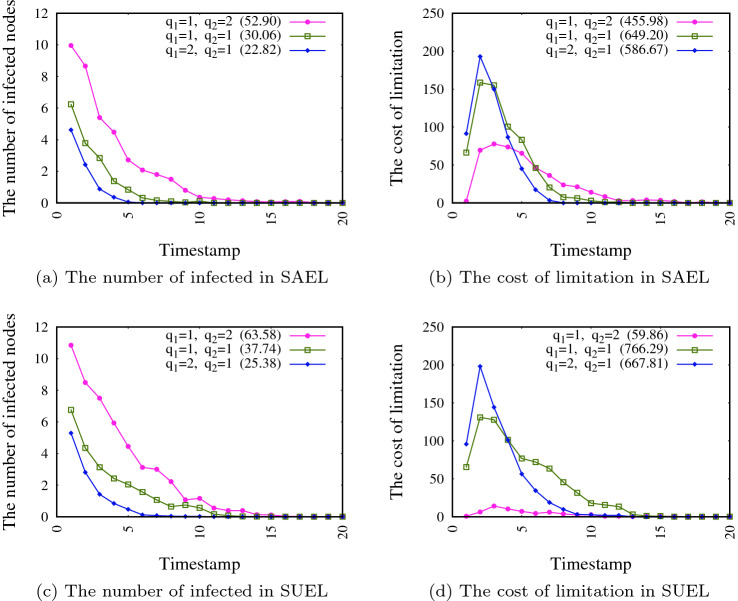
The number of infected individuals and cost of limitation in different timestamps for network SN1

**Fig. 7:**
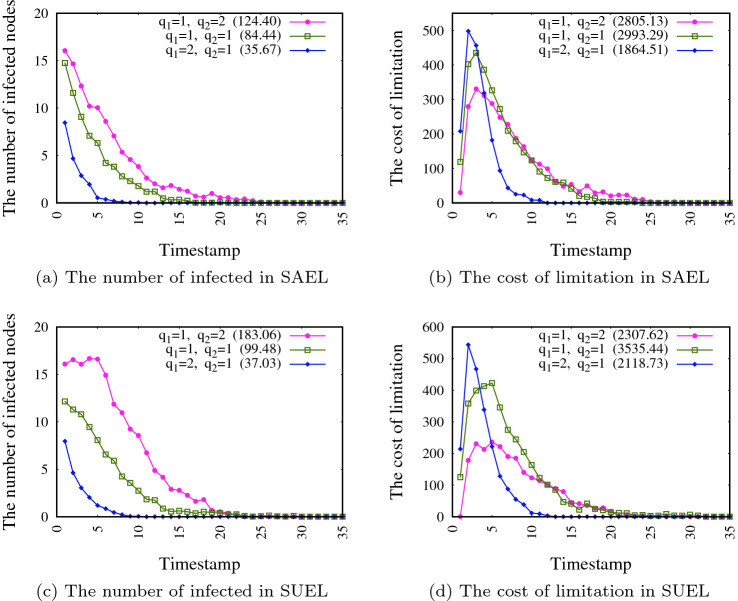
The number of infected individuals and cost of limitation in different timestamps for network SN2

The number of infected individuals and the cost of edge limitation in different timestamps is evaluated in this experiment. The results for both SAEL and SUEL for each of the networks SN1 and SN2 are shown in Figs. 6 and 7, respectively. In this experiment, we consider again optional relative importance ($q_1$ and $q_2$) to obtain different weights for $f_1$ and $f_2$ to evaluate the impact of these values on the number of infected individuals and limitation costs in different timestamps. In these figures, the total number of infected individuals and the limitation cost during the whole pandemic period are shown in parenthesis in the legend of charts. The results suggest that higher weight values for $f_1$ (green lines) lead to a smaller number of infected people in each timestamp, which results in less pressure in some timestamps. However, it also results in a strict limitation (and high costs) in some timestamps (see for example the costs in timestamps 1–3 in Fig. 6b, d). Also, as can be seen from the figures, considering an equal weight for each of the two objectives leads to a moderate number of infected individuals and the limitation costs is not too high in each timestamp, but the total costs are the highest because avoiding strict limitations (due to the high cost in each timestamp), the spreading period takes more time and leads to a higher total cost of limitation. A comparison of Figs. 6 and 7 shows that the spreading process takes longer when the size of network is greater.

### Experiment 4

**Fig. 8:**
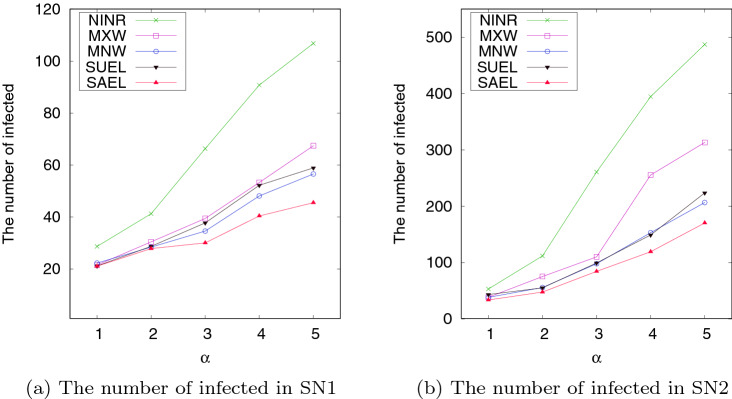
The number of infected for different values of $\alpha$

The performance of the proposed methods in terms of minimizing the total number of infected individuals for different values of $\alpha$ is compared to other methods in this section. To make a fair comparison where each method results in the same cost, SAEL is first applied to determine the limitation factor of the edges during the spreading period; the cost of limitation in each timestamp is stored. Then, this stored value is considered as a threshold in each timestamp for all methods to determine the number of edges which can be limited in the timestamp. The total number of the infected individuals at the end of the spreading process is reported in this experiment. This experiment is repeated for different values of $\alpha$ (the number inter-community neighbours of a node when the nodes commute to another community). The results obtained by this experiment are shown in Fig. 8. It can be seen that SAEL outperforms the other methods for the same cost, especially for higher values of $\alpha$, in which case edge limitation plays a more important role to restrain virus spread. The results in the figure also show that SUEL, which is internal relationship agnostic, has good performance compared to methods such as SAEL, MXW and MNW, which are aware of the internal relationships.

## Conclusion

Minimizing the interaction between individuals and communities is an approach to contain the spread of a virus, however, it comes at a cost. In this paper, a framework to model the spreading process of a virus in intra- and intra-community interactions was proposed. The problem of limiting interactions was defined as a multi-objective problem where the aim is to achieve a trade-off between the number of infected individuals and the cost of limitations. Then, PSO was applied to find an optimal solution of the problem under two different scenarios. The experimental results suggest that the performance of the proposed methods compared favourably to simple heuristics. Future work may focus on modelling the problem using large-scale networks and designing different strategies to determine the limitation factor of each edge. How to model interactions when little information is available or taking into account uncertainty in the interactions can also be an issue for future work. Finally, all individuals in this paper are considered the same (homogeneous population); yet, different individuals may have different levels of health risk. Modelling individuals with a different level of risk and minimizing infection based on the individuals’ level of risk could also be considered.
